# Supplementary figures and images for: High SARS-CoV-2 load in the nasopharynx of patients with a mild form of COVID-19 is associated with clinical deterioration regardless of the hydroxychloroquine administration
Source: PLoS One. 2021 Jan 29;16(1):e0246396. doi: 10.1371/journal.pone.0246396 (PMC7846025; doi:10.1371/journal.pone.0246396)

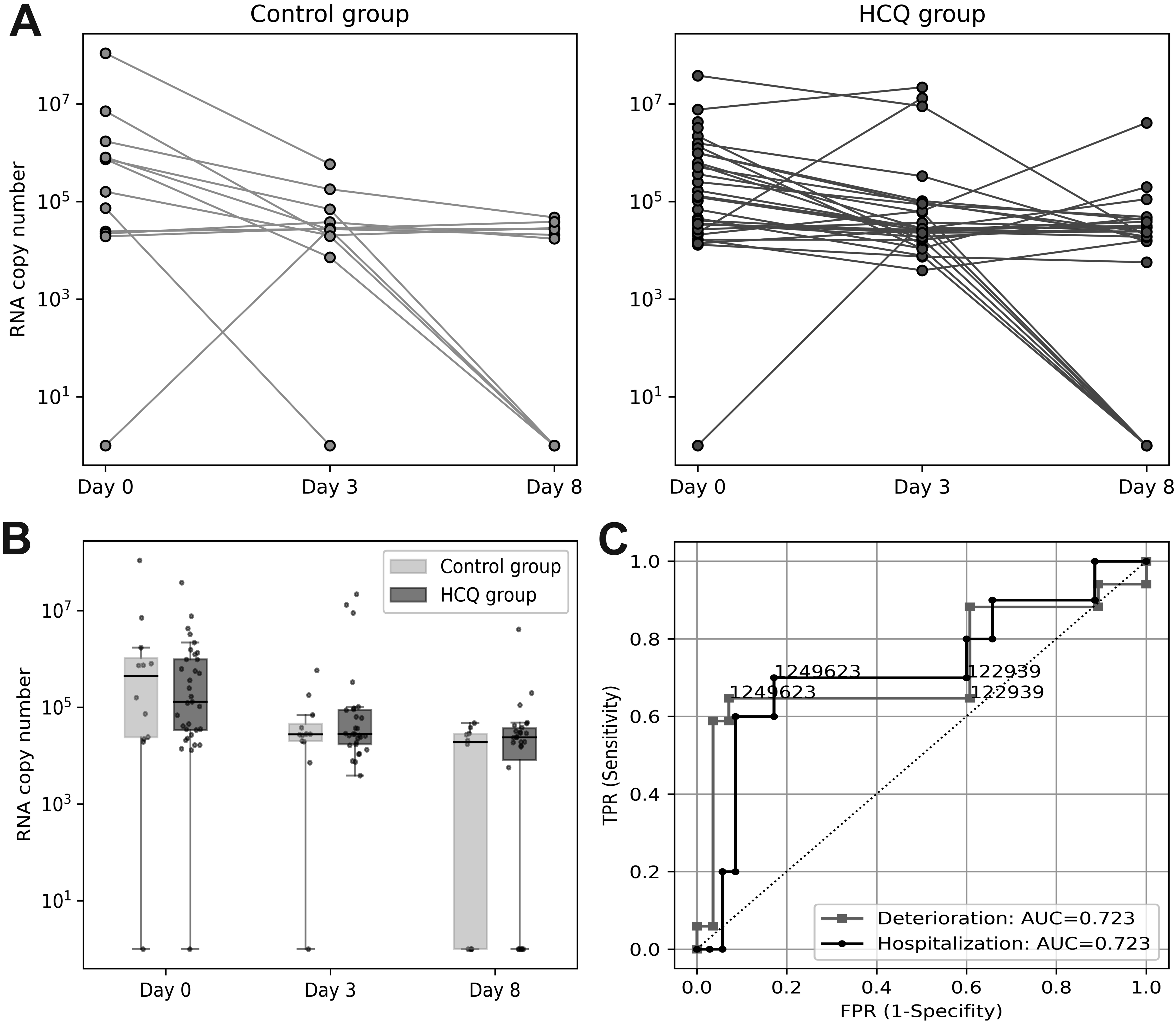

Supplement: S1 Fig — Individual dynamics (A) and intergroup comparison (B) of measured RNA copy number. Each curve represents a patient. To use the logarithmic scale, the exact 0 was replaced with 1 (100). Shown are ROC-curves and corresponding area under the curve (AUC) values reflecting the sensitivity and specificity of different viral RNA load values used to predict outcomes with clinical deterioration or hospitalization (C). (TIF) [file pone.0246396.s001.tif]
