# Supplementary material for: High SARS-CoV-2 load in the nasopharynx of patients with a mild form of COVID-19 is associated with clinical deterioration regardless of the hydroxychloroquine administration
Source: PLoS One. 2021 Jan 29;16(1):e0246396. doi: 10.1371/journal.pone.0246396 (PMC7846025; doi:10.1371/journal.pone.0246396)
Supplement: Attachment — Submitted filename: Comments on paper Helena Vasilieva.pdf [file pone.0246396.s002.pdf]

Comments on paper "Hydroxychloroquine has no effect on SARS-CoV-2 load in nasopharynx of patients with mild form of COVID-19"

Dr. Helena Vasilieva

General comments

This is a nice paper on a small number of non-randomised patients with clinically mild, virologically-diagnosed COIVD-19. The paper demonstrates that at the dose of hydroxychloroquine (HCQ) used there was no apparent effect on the viral load compared to a smaller group of controls.

There is scope to improve the paper and specifics are listed below.

Abstract

We need to see the dose of HCQ recommended in Russia at the time of the study and the dose given to patients in this report.

Need to see the size of each group and it should be stated here that the study was a non-randomised, observational study.

The last sentence should be modified to say at the dose used, HCQ…….

Introduction

The authors must mention the Recovery and Solidarity trials which showed that chloroquine and HCQ, respectively, had no effect in severe COVID-19 disease. This paper has also just been published in JAMA and the authors may wish to cite this as well:

Self et al. National Heart, Lung, and Blood Institute PETAL Clinical Trials Network. Effect of hydroxychloroquine on clinical status at 14 days in hospitalized patients with COVID-19: a randomized clinical trial. *JAMA*. Published online November 9, 2020.

They must also mention several studies of viral kinetics on COVID-19 to give readers an idea of how viral load varies over time; the focus should be on patients with mild disease and asymptomatic individuals.

We must know the dose of HCQ used in Russia as treatment and prophylaxis.

Methods

How were the patients recruited ?

Were there no formal inclusion or exclusion criteria ?

The authors have defined clinical deterioration but what were the clinical indications for hospitalisation ? The authors may wish to refer to the WHO classification of COVID-19 severity.

For the viral loads, the authors talk of taking into account the volume of viral transport medium so I am expecting to see viral load/unit volume but this is not reported. Please clarify.

We must know the dose of HCQ used in this study.

The authors should mention here how they chose a viral load of 10^6 to use as a marker to differentiate those with and without clinical deterioration.

I have never heard of a continuity correction for a Mann Whitney U test. Please remove.

Results & Discussion

Clinicians will want to know that all patients had virologically-proven COVID-19. This is not clear from the results as some patients did not have a detectable RNA load. Please clarify.

It is unclear to me regarding the patient with a zero viral load when the authors say the lower limit of detection with the qPCR is 1000 copies.

Line 147. The authors speculate that a RNA viral load of 10^4 may be associated with clinical recovery but no clinical data are shown.

In describing the viral dynamics, I suggest the authors use such Figures as:

Individual viral loads over time (raw data & log transformed) with illness day on the X axis using different colours for the control and HCQ groups. This will give a much better idea of viral dynamics. If this is too busy, then show two graphs – control & HCQ.

Box plots of viral load using the raw data on D0, 3 & 8 with one plot for the control group and another for the HCQ group. This will give a visual impression to support the data in Table 3.

The authors suggest a high viral load is associated with clinical deterioration and hospital admission. This is an important finding. Have other authors found this ?

Hypertension is mentioned as a significant factor but did the subjects have any other comorbidities ?

Were any patients on statins ?

Although the study is small with limited statistical power, the authors may wish to use logistic regression to look at several factors for clinical deterioration and hospital admission. Similarly, for the viral load over time between the two groups, repeated measures ANCOVA (log transformed data) or mixed effects modelling could be used to compare the two groups.

Conclusion

All the authors can say here is that their small study of mild disease did not show an apparent effect on viral load of HCQ at the dose used. Perhaps the dose was too low ? They cannot extrapolate their findings to primary prophylaxis so this should be removed.
