# Supplementary material for: High SARS-CoV-2 load in the nasopharynx of patients with a mild form of COVID-19 is associated with clinical deterioration regardless of the hydroxychloroquine administration
Source: PLoS One. 2021 Jan 29;16(1):e0246396. doi: 10.1371/journal.pone.0246396 (PMC7846025; doi:10.1371/journal.pone.0246396)
Supplement: Attachment — Submitted filename: Komissarov et al_response to reviewers.doc [file pone.0246396.s004.doc]

All authors would like to thank the Academic Editor for the suggested text edits. The amendments introduced are listed below:

**Response to the Academic Editor’s comments:**

**Abstract:**

*“Abstract*

*I suggest you change the text to something like this:*

*In the current non-randomized, observational study we evaluated the SARS-CoV-2 RNA in nasopharynx swabs from infected patients 7-10 days post symptoms with clinically mild disease and compared the viral RNA load dynamics between patients receiving HCQ (200 mg twice per day according to the Ministry of Health of Russian Federation treatment instructions, n = 33) and a control group without antiviral pharmacological therapy (n = 12).”*

The text was modified according to the comment (Page 2, Line 28).

*“Introduction*

*Please change this because ‘highly controversial’ does not apply to all studies:*

*From:*

*Furthermore, use of chloroquine and HCQ for SARS-CoV-2 patients with severe disease has been reported, but the results of these studies are highly controversial [4-10]. While there are not enough clinical data to support the use of HCQ, several countries including Russia have included HCQ in treatment protocols for infected patients and for prophylaxis.*

*To:*

*Early in the endemic, there were insufficient clinical data to support a particular dose of HCQ so several countries like Russia recommended different HCQ doses as treatment for infected patients and as prophylaxis.*

*Initial results suggesting that HCQ and CQ had antiviral activity and were beneficial (Lammers, Gao Gautret) were not confirmed in the large Solidarity and Recovery randomized trials of severe COVID-19 and early treatment of mild COVID-19 (Mitja et al, Skipper et al); these trials used higher HCQ doses than the one recommended in Russia.*

*Please amend your referencing accordingly.”*

Corresponding edits and referencing were introduced in accordance with suggested text variant (Page 3, Lines 43-49).

*“Methods*

*Line 66: change to: while 12, who declined HCQ, represented a control group.”*

The corresponding sentence was modified according to the comment (Page 4, Line 67).

*“Conclusion*

*I suggest changing line 243 to:*

*At the dose used in this study in mildly ill patients who were symptomatic for at least one week, HCQ did not accelerate viral clearance compared to no HCQ.”*

The text was modified according to the comment (Page 12, Lines 238-240).
